# Lateral lymph node metastasis in a patient with T1 upper rectal cancer treated by lateral lymph node dissection: a case report and brief literature review

**DOI:** 10.1186/s40792-017-0366-3

**Published:** 2017-08-23

**Authors:** Hiroyuki Tanishima, Masamichi Kimura, Toshiji Tominaga, Shinji Iwakura, Yoshihiko Hoshida, Tetsuya Horiuchi

**Affiliations:** 1 Department of Surgery, National Hospital Organization, Osaka Minami Medical Center, 2-1 Kidohigashimachi, Kawachinagano, Osaka Japan; 2 Department of Pathology, National Hospital Organization, Osaka Minami Medical Center, 2-1 Kidohigashimachi, Kawachinagano, Osaka 586-8521 Japan

**Keywords:** Lateral lymph node metastasis, T1 upper rectal cancer, Lateral lymph node dissection, Perioperative examination

## Abstract

**Background:**

Lateral lymph node (LLN) metastasis may occur in patients with advanced rectal cancers of which the lower margins are located at or below the peritoneal reflection. However, LLN metastasis from a T1 rectal cancer is rare. Here, we report a case of LLN metastasis from a T1 upper rectal cancer that was successfully treated by sequential LLN dissection.

**Case presentation:**

A 56-year-old man was referred to our hospital for the treatment of a T1 upper rectal cancer. We performed a laparoscopic low anterior resection. Histological examination showed a moderately differentiated adenocarcinoma with submucosal layer invasion; the invasion depth was classified as head invasion, without vessel or lymph duct invasion. Tumor budding was classified as grade 1. A total of six lymph nodes were harvested, and no lymph node metastases were detected. The postoperative course was uneventful. At 6 months after surgery, however, the serum carcinoembryonic antigen levels were elevated, and abdominal computed tomography (CT) revealed swollen lymph nodes in the right internal and common iliac artery area. Positron emission tomography with CT revealed hot spots in the same lesions. A retrospective re-evaluation of the preoperative CT images revealed no apparent swollen lymph nodes; however, an unusual soft tissue area was detected around the right internal iliac artery. A right LLN dissection was performed. Fifteen lymph nodes were resected, and histologically, metastases of adenocarcinoma were identified in 3 nodes. The postoperative course was again uneventful. The patient was given 12 cycles of adjuvant chemotherapy with FOLFOX (fluorouracil, leucovorin, and oxaliplatin). The patient remains healthy and with no signs of recurrence at 30 months after the second surgery.

**Conclusions:**

LLN metastasis occurs very rarely in patients with T1 upper rectal cancer and no risk factors for lymph node metastasis; however, a careful perioperative examination of the LLN should be performed. In cases involving LLN metastasis, a LLN dissection may be a therapeutic option if performed with curative intent.

## Background

The standard treatment for T1 rectal cancer is total mesorectal excision (TME) without preoperative chemoradiotherapy in Western countries and TME without lateral lymph node (LLN) dissection in Japan [1–3]. LLN metastases are detected in approximately 15% of patients with rectal cancer; however, LLN metastases from T1 rectal cancers are rare [4–6]. Here, we report a case of LLN metastasis from a T1 upper rectal cancer that was successfully treated by sequential LLN dissection.

## Case presentation

**Fig. 1:**
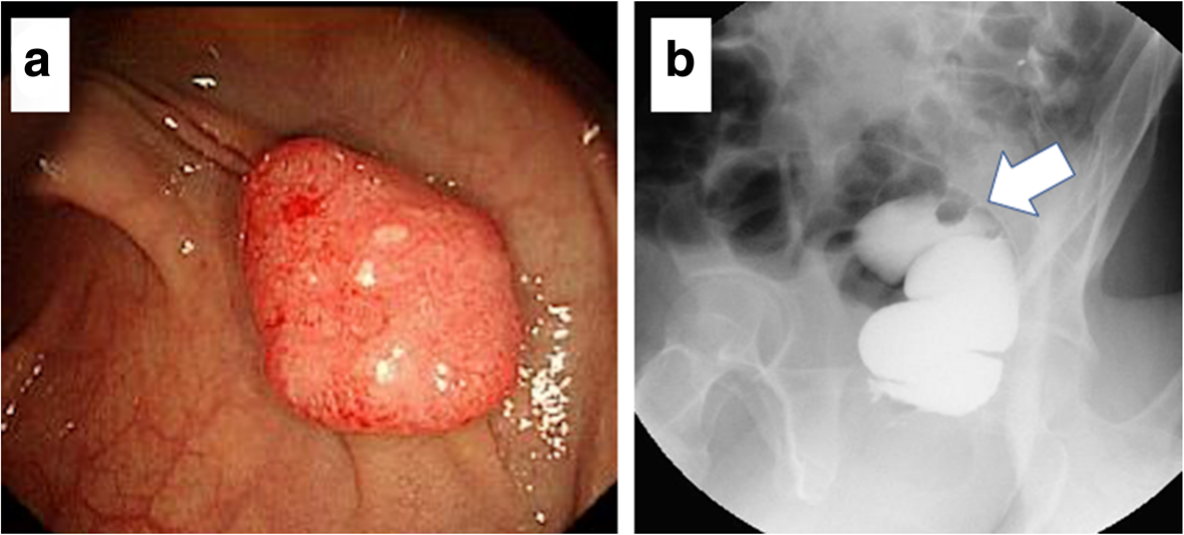
Endoscopic and proctographic findings. **a** Colonoscopy revealed a pedunculated-type tumor measuring 1.5 cm × 1.0 cm. **b** Proctographic examination revealed a filling defect in the upper rectum (*arrow*)

**Fig. 2:**
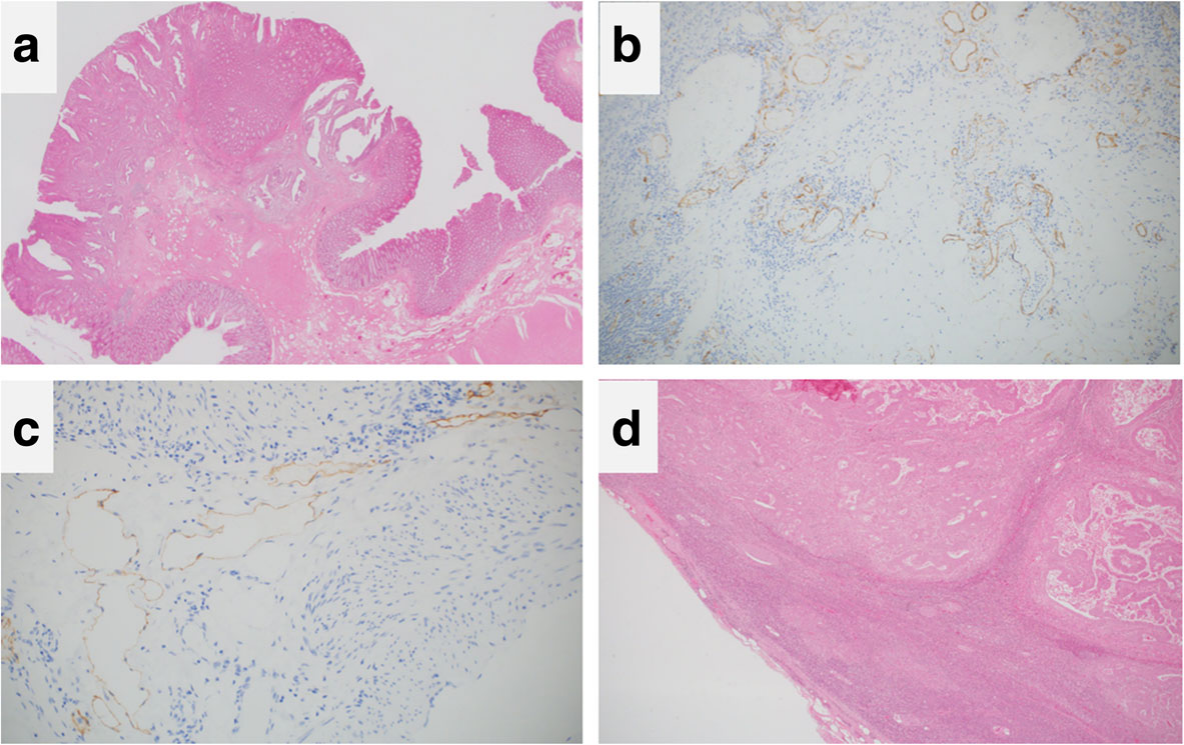
Histological examination showed a moderately differentiated adenocarcinoma that had invaded the submucosal layer; the invasion depth was classified as a head invasion (**a**). Immunohistochemical staining for CD34 (**b**) and D2-40 (**c**) revealed no infiltration of the vessels or lymph ducts. **d** The resected lateral lymph nodes confirmed a metastasis of moderately differentiated adenocarcinoma

A 56-year-old man was referred to our hospital for the treatment of rectal cancer. The patient was otherwise healthy, without significant previous or current medical problems. His serum carcinoembryonic antigen (CEA) and carbohydrate antigen 19-9 levels were 1.8 ng/mL (normal, <3.4 ng/mL) and 7.7 U/mL (normal, <37 U/mL), respectively. Colonoscopy revealed a pedunculated-type tumor measuring 1.5 cm × 1.0 cm in the upper rectum, 12 cm from the anal verge, and proctographic examination revealed a filling defect in the upper rectum (Fig. 1a, b). Contrast-enhanced computed tomography (CT) revealed no swollen lymph nodes or distant metastases. We diagnosed the rectal cancer as T1N0M0 stage I and performed a laparoscopic low anterior resection. Regarding the extent of lymph node dissection, a division of the superior rectal artery root was performed without LLN dissection. A histological examination revealed a moderately differentiated adenocarcinoma that had invaded the submucosal layer (T1); the invasion depth was classified as a head invasion, without vessel or lymph duct invasion (Fig. 2a–c). Tumor budding was classified as grade 1. A total of six lymph nodes were harvested, and no lymph node metastases were detected. The postoperative course was uneventful.

**Fig. 3:**
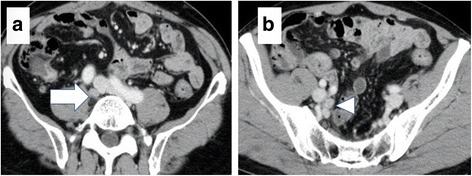
Contrast-enhanced computed tomography. Images show swollen lymph nodes **a** in the right common iliac artery area (*arrow*) and **b** right internal iliac artery area (*arrowhead*)

**Fig. 4:**
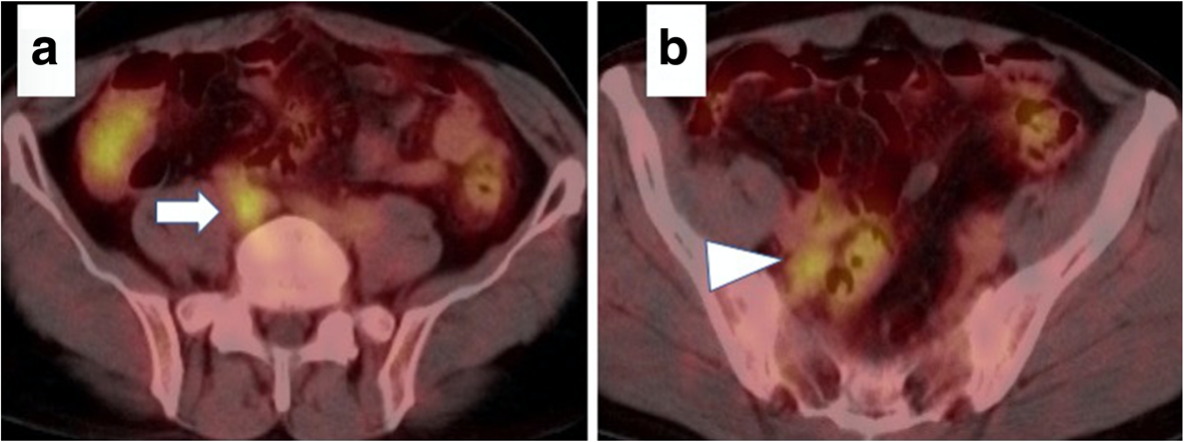
Positron emission tomography. Images show hot spots **a** in the right common iliac artery area and **b** right internal iliac artery area

**Fig. 5:**
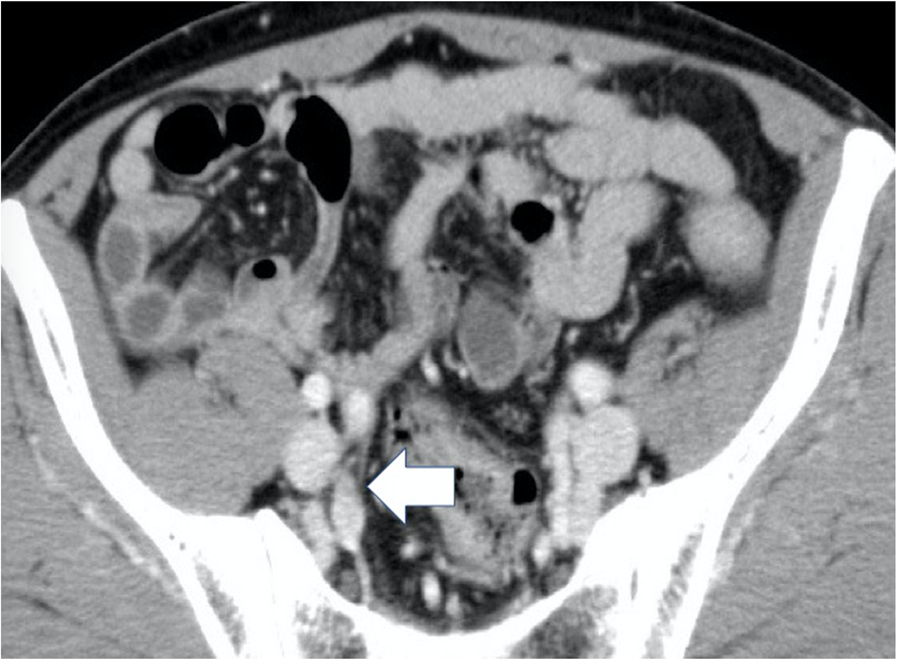
Contrast-enhanced computed tomography performed prior to initial surgery. The image shows an unusual area of soft tissue around the right internal iliac artery (arrow)

Six months after the operation, however, the patient’s serum CEA levels increased to 7.0 ng/mL. Abdominal CT revealed swollen lymph nodes in the right common and internal iliac artery area (Fig. 3a, b). Positron emission tomography (PET) with CT revealed hot spots (SUV_max_, 5.3) in the same lesions (Fig. 4a, b). No other metastases were observed. Accordingly, we retrospectively re-evaluated the preoperative CT images. Although we detected no apparent swollen lymph nodes, we observed an unusual soft tissue area around the right internal iliac artery (Fig. 5). The preoperative diagnosis was an LLN metastasis localized in the right pelvic area, and an open unilateral LLN dissection of the right common iliac, internal iliac, and obturator nodes was performed. The branches of the right internal iliac vessels, including the superior vesical and obturator vessels, were ligated and divided at their origins with resected lymph nodes; however, the internal iliac artery and pelvic nerve plexus were preserved. Histologically, 15 lymph nodes were resected; of these, 3 (2 in the proximal internal iliac node and 1 in the common iliac node) contained metastases of adenocarcinoma (Fig. 2d). The postoperative course was uneventful. The patient was given 12 cycles of adjuvant chemotherapy with FOLFOX (fluorouracil, leucovorin, and oxaliplatin). He remains healthy without signs of recurrence at 30 months after the second surgery.

### Discussion

The course of the patient described in this report suggests two important clinical issues. First, LLN metastasis can occur in a patient with T1 upper rectal cancer and no risk factors for lymph node metastasis; second, sequential LLN dissection is useful for the treatment of this condition.

With regard to the first point, LLN metastasis occurs in 7.7–28.8% of patients with T3–T4 lower rectal cancer [4]. In contrast, the reported incidence of LLN metastasis among patients with T1 lower rectal cancer is only 0.9% [4]. To date, only four cases of isolated LLN metastasis from a lower rectal cancer have been reported [7–10], and to our knowledge, ours is the first case of LLN metastasis in a patient with T1 upper rectal cancer. The incidence of lymph node metastasis from a T1 colorectal cancer is approximately 15% [3]. Additional major surgery is recommended after a successful endoscopic excision if the tumor has at least one risk factor for lymph node metastasis, including an invasion depth ≥1000 μm from the muscularis mucosa, lymphatic and vascular invasion, non-well or moderately differentiated adenocarcinoma, and high-grade budding [3]. Table 1 presents a review of the five reported cases (including this case) of isolated LLN metastasis from T1 rectal cancer. Three of the five cases had risk factors for lymph node metastasis [8–10]; in contrast, our case had no identified risk factors and involved a pedunculated-type tumor with head invasion that could be managed by endoscopic treatment alone [11].

**Table 1:** Review of four reported cases (including the present case) of isolated LLN metastasis from a T1 rectal cancer

Case	Year	Author	Age (years)	Sex	Location of primary tumor	Risk factors of lymph node metastasis				
						Depth of invasion (μm)	Histological type	ly	v	Budding grade
1	2008	Hara	61	Male	Lower	–	Well	0	0	–
2	2010	Yamaguchi	67	Female	Lower	3000	Well to moderately	0	0	–
3	2013	Sueda	41	Female	Lower	–	Moderately	0	Positive	–
4	2016	Ogawa	35	Female	Lower	3000	Moderately	Positive	0	–
5	2017	Ours	56	Male	Upper	Head invasion	Moderately	0	0	1

*LLN* lateral lymph node, not described

In general, enhanced CT is used as a preoperative imaging tool for rectal cancer. Currently, the European Society for Medical Oncology guidelines recommend pelvic magnetic resonance imaging (MRI) for the initial staging of rectal cancer because this modality is highly accurate for determining localization, clinical T and N stages, and potential circumferential resection margins [1]. The diagnostic sensitivity and specificity of MRI for lymph node metastasis are 77 and 71%, respectively [12]. Additionally, the usefulness of diffusion-weighted imaging (DWI)-MRI has recently been reported [13–15]. The accuracy of DWI-MRI is better than that of both CT and ^18^FDG-PET (86.6 vs. 76.0% and 78.3 vs. 69.9%, respectively) [14, 15]. In our case, we considered the patient to be metastasis-negative, based on enhanced CT findings. The LLN metastasis was revealed only 6 months after the initial surgery, when an unusual soft tissue area around the right internal iliac artery was retrospectively detected on preoperative CT images. Based on these two factors, it is possible that the LLN metastasis existed at the time of initial surgery and could have been diagnosed by DWI-MRI.

With regard to the second point about the therapeutic usefulness of sequential LLN dissection, the status of the LLN has not been fully established. In Japan, LLN metastasis is considered a local disease [16], and prophylactic LLN dissection is recommended [3]. This type of dissection does not appear to increase morbidity, mortality, or sexual dysfunction [17, 18]. In Western countries, LLN is categorized as a distant metastasis, and preoperative chemoradiotherapy, rather than prophylactic LLN dissection, has been administered to patients with advanced rectal cancers [l, 2]. However, therapeutic LLN dissection is recommended at the time of primary tumor resection for cases with clinically evident LLN metastasis, assuming that curative resection can be achieved [19]. Extended LLN dissection is recommended due to favorable oncologic outcomes in this situation [6, 7]. Table 2 lists the treatments and outcomes of the five cases of isolated LLN metastasis from T1 rectal cancer. In four cases, extended LLN dissection was performed [7–10]. We disagree with the LLN dissection with skeletonizing of the branches of the internal iliac vessels; however, we think a routine en bloc resection of the internal iliac vessels is not necessary when the metastatic tumors do not invade or adhere firmly to the internal iliac vessels because there is the possibility of increasing the risk of complications. In our case, the internal iliac vessels were preserved, while the branches of the internal iliac vessels, including the superior vesical and obturator vessels, were resected with the lymph nodes. Bilateral LLN dissection has greater benefits on reducing local recurrence than unilateral LLN dissection [20]; however, in all four cases, the patients with isolated LLN metastases from early rectal cancers were treated successfully via unilateral LLN dissection in the affected side [7–10]. With regard to adjuvant chemotherapy, two patients refused chemotherapy after LLN dissection [7, 9].

**Table 2:** Treatments and outcomes of the four reported cases of isolated LLN metastasis from a T1 rectal cancer

Case	Time after 1st surgery (months)	Treatment	Bilateral or unilateral LLND	Chemotherapy after LLLD	Outcome and time after LLND (months)
1	22	Extended LLND	Unilateral	None	Alive
2	Synchronous	Extended LLND	Unilateral	5-Fluorouracil	Alive
3	6	Extended LLND	Unilateral	None	Alive
4	Synchronous	Extended LLND	Unilateral	Tegafur-uracil + leucovorin	Alive
5	6	LLND	Unilateral	FOLFOX	Alive

*LLND* lateral lymph node dissection, *FOLFOX* fluorouracil, leucovorin, and oxaliplatin

## Conclusions

LLN metastasis can occur in patients with T1 upper rectal cancer and no risk factors for lymph node metastasis, and sequential LLN dissection is useful for the treatment of this condition. Although LLN metastasis occurs very rarely in this patient population, a careful perioperative evaluation of the LLN should be performed. In cases involving LLN metastasis, LLN dissection could be considered a therapeutic option if performed curatively.

## Abbreviations

CEA: Carcinoembryonic antigen
CT: Computed tomography
DWI: Diffusion-weighted imaging
LLN: Lateral lymph node
MRI: Magnetic resonance imaging
PET: Positron emission tomography
